# High-resolution genetic and physical mapping reveals a peanut spotted wilt disease resistance locus, *PSWDR-1*, to Tomato spotted wilt virus (TSWV), within a recombination cold-spot on chromosome A01

**DOI:** 10.1186/s12864-025-11366-7

**Published:** 2025-03-06

**Authors:** Dongliang Wu, Chuanzhi Zhao, Walid Korani, Ethan A. Thompson, Hui Wang, Gaurav Agarwal, Jake C. Fountain, Albert Culbreath, C. Corley Holbrook, Xingjun Wang, Josh P. Clevenger, Baozhu Guo

**Affiliations:** 1 Department of Plant Pathology, University of Georgia, Tifton, GA USA; 2 Institute of Crop Germplasm Resources, Shandong Academy of Agricultural Sciences, Shandong Provincial Key Laboratory of Crop Genetic Improvement, Ecology and Physiology, Jinan, China; 3 USDA-ARS Crop Genetics and Breeding Research Unit, Tifton, GA USA; 4 HudsonAlpha Institute for Biotechnology, Huntsville, AL USA; 5 Department of Plant Biology, Michigan State University, East Lansing, MI USA; 6 Department of Plant Pathology, University of Georgia, Griffin, GA USA

**Keywords:** *Arachis hypogaea*, Linkage and physical mapping, TSWV, Peanut spotted wilt disease resistance locus, Recombination cold spot

## Abstract

**Background:**

Peanut (*Arachis hypogaea* L.) is a vital global crop, frequently threatened by both abiotic and biotic stresses. Among the most damaging biotic stresses is Tomato spotted wilt virus (TSWV), which causes peanut spotted wilt disease resulting in significant yield loss. Developing TSWV-resistant cultivars is crucial to new cultivar release. Previous studies have used a subset of the “S” recombinant inbred line (RIL) population derived from SunOleic 97R and NC94022 and identified quantitative trait loci (QTLs) for resistance to TSWV. These studies utilized different genotyping techniques and found large consistent genomic regions on chromosome A01. The objective of this study was to fine map the QTL and identify candidate genes using the entire population of 352 RILs and high-density, high-quality peanut SNP arrays.

**Results:**

We used both versions of the peanut SNP arrays with five years of disease ratings, and successfully mapped the long-sought peanut spotted wilt disease resistance locus, *PSWDR-1*. QTL analyses identified two major QTLs, explaining 41.43% and 43.69% of the phenotypic variance within 3.6 cM and 0.28 cM intervals using the peanut Axiom_*Arachis*-v1 and Axiom_*Arachis*-v2 SNP arrays, respectively, on chromosome A01. These QTLs corresponded to 295 kb and 235 kb physical intervals. The unique overlap region of these two QTLs was 488 kb. A comparison of the genetic linkage map with the reference genome revealed a 1.3 Mb recombination “cold spot” (11.325–12.646 Mb) with only two recombination events of RIL-S1 and RIL-S17, which displayed contrasting phenotypes. Sequencing of these two recombinants confirmed the cold spot with only five SNPs detected within this region.

**Conclusions:**

This study successfully identified a peanut spotted wilt disease resistance locus, *PSWDR-1*, on chromosome A01 within a recombination “cold spot”. The *PSWDR-1* locus contains three candidate genes, a TIR-NBS-LRR gene (*Arahy.1PK53M*), a glutamate receptor-like gene (*Arahy.RI1BYW*), and an MLO-like protein (*Arahy.FX71XI*). These findings provide a foundation for future functional studies to validate the roles of these candidate genes in resistance and application in breeding TSWV-resistant peanut cultivars.

**Supplementary Information:**

The online version contains supplementary material available at 10.1186/s12864-025-11366-7.

## Background

Tomato spotted wilt virus (TSWV) is a major pathogen affecting peanut (*Arachis hypogaea* L.) production in the southeastern United States, particularly in Georgia, Florida, and Alabama. TSWV caused annual losses of approximately $12.3 million in the U.S. peanut industry [1]. The virus is primarily transmitted by thrips, with tobacco thrips (*Frankliniella fusca*) being the primary vector for peanut inoculation. While chemical pesticides are commonly used to control thrips and reduce TSWV incidence, they are costly and pose environmental risks. An alternative and sustainable strategy is the utilization of resistant cultivars in an integrated pest management system to manage TSWV in peanuts.

A breeding line NC94022 exhibited the highest field resistance to TSWV [2]. This resistance likely inherited from its parent PI576638, a *hirsuta*-type line from Mexico [3]. In 2012, a major quantitative trait locus (QTL) associated with the resistance was first identified using a recombinant inbred line (RIL) mapping population, known as the “S” population, from a cross between SunOleic 97R (susceptible to TSWV) and NC94022 [4]. Qin et al. (2012) used a subset of 190 RILs and 181 polymorphic simple sequence repeat (SSR) markers and identified a major TSWV resistance QTL (*qTSWV*2 on chromosome A01), which explained 35.8% of the phenotypic variance. In 2016, this finding was validated by genotyping the entire “S” population of 352 RILs using 248 polymorphic SSR markers and multi-year field phenotypes [5], which reported a major QTL (*qTSW*-T13_A01_4) with a phenotypic variance explained (PVE) of 29.14%. There were two more reports from a different research group mapping the resistance QTL of NC94022, using SSR markers and a mapping population derived from a cross of Florida-EP™ ‘113’ (a resistant line derived from NC94022) and Georgia Valencia (susceptible to TSWV) [6, 7]. Both reports identified a major QTL on chromosome A01 that had smaller PVE and near the pericentromeric region.

High quality markers were needed for improved marker density for increased mapping resolution. With the completion of peanut genome sequencing projects for diploid and tetraploid peanut [8–10] and availability of genomic resources and tools, the first version of a peanut large-scale (58K) SNP genotyping array ‘Axiom_*Arachis-*v1’ was developed. This array included 44,501 SNPs from tetraploid genotypes and 13,732 SNPs from diploid genotypes [11, 12]. Since then, genetic trait mapping in peanut made a great progress in genotyping and accuracy in QTL analysis of complex traits like pod weight, seed weight and fresh seed dormancy [13–15]. An improved version of the peanut SNP array, the 48K ‘Axiom_Arachis-v2’, was developed by Clevenger et al. (2018) [16]. This array incorporates 11,516 SNPs from the first version, 1,674 haplotype-based SNPs from contrasting sub-genome-specific sequences, and 28,218 newly identified SNPs from the re-sequencing of 21 tetraploid accessions including three parents of the “T” and the “S” populations [4]. This updated array is a valuable resource for constructing high-resolution genetic linkage maps in peanuts.

In 2019, Agarwal et al. [17] employed whole-genome resequencing (WGRS) to genotype a subset of the “S” population and constructed the first bin-based linkage map, resulting in identification of three QTLs for resistance to TSWV, co-localized on chromosome A01 with two potential candidate genes, a chitinase gene cluster and an NBS-LRR disease resistance gene cluster [17]. Collectively, these reports support the presence of a major QTL linked to TSWV resistance on chromosome A01 in NC94022 [4–7, 17].

The goal of the present study was to use all the resources available, including the entire “S” population of 352 RILs and the improved high-quality SNP array, the 48K Array ‘Axiom_Arachis-v2’ to fine-map the peanut spotted wilt disease resistance locus (*PSWDR*−1) on the chromosome A01 and to identify the potential candidate genes for further characterization of the resistance mechanisms. Interestingly, a recombination cold-spot was identified where the *PSWDR*−1 was located. The recombination cold-spots are genomic regions with infrequent recombination events, crucial for maintaining genetic stability by preserving essential genetic markers and desirable traits. These regions facilitate accurate QTL mapping of traits of interest. Traits within cold-spots are inherited together, retaining favorable allele combinations. Understanding these regions helps breeders design effective strategies, improving crop traits and breeding efficiency. The haplotypes in this cold-spot revealed that there were only two lines, RIL-S1 and RIL-S17, out of the 352 RILs with genetic crossover within this cold-spot. RIL-S1 and RIL-S17 have contrasting phenotypes and the physical map by sequencing of these two lines supports this recombination cold-spot. Within this locus, there were three candidate genes including an NBS-LRR disease resistance gene, a glutamate receptor, and an MLO-like protein.

## Results

### Phenotypic variation

**Fig. 1:**
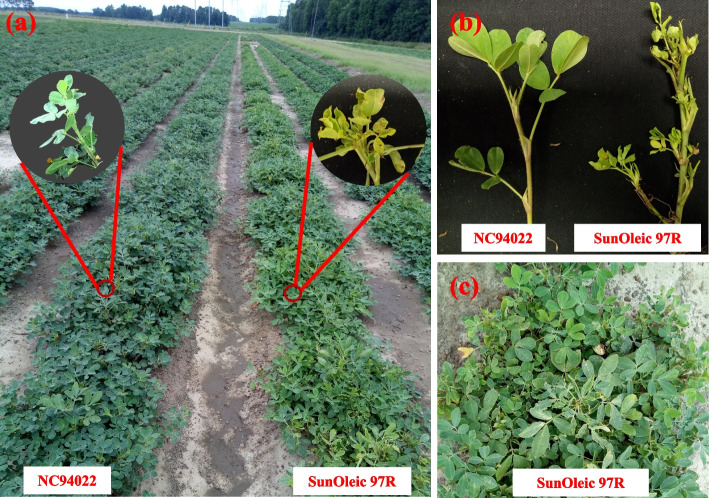
Tomato spotted wilt virus (TSWV) disease symptoms in NC94022 and SunOleic 97R in the field. **a** Rows of NC94022 and SunOleic 97R in the field. **b **Comparison of leaves. **c **SunOleic 97R TSWV infected plants

**Fig. 2:**
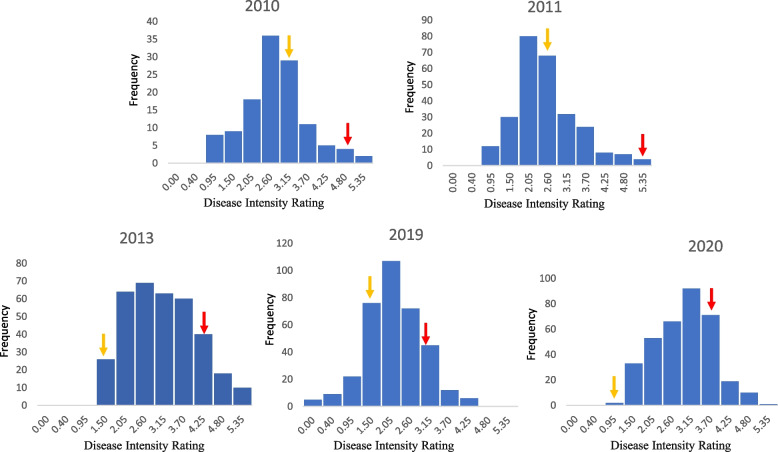
Distribution of Tomato spotted wilt virus (TSWV) disease intensity rating through the years. The horizontal axis shows the disease intensity rating and the vertical axis indicates the disease rating frequency whereas the disease severities of the parents were indicated by the colored arrows, yellow for the resistant parent NC94022 and red for the susceptible parent SunOleic 97R

Peanut TSWV disease severity was evaluated in the field over five years (2010, 2011, 2013, 2019, and 2020). The two parental lines showed a considerable difference in resistance. The resistant parent, NC94022, had an average disease rating of 1.3 with standard deviation of 0.6, while the susceptible parent, SunOleic 97R, had an average rating of 4.3 with standard deviation of 0.6 (Fig. 1). The RIL population exhibited significant phenotypic variation in TSWV disease ratings across multiple years (Table 1). Some RILs consistently exhibited low disease ratings like the resistant parent, while others showed moderate to high susceptibility across all five years. The distribution of disease severity ratings was continuous and normal, as indicated by the Shapiro–Wilk (w) test (Table 1; Fig. 2).

**Table 1:** Statistical analysis of disease severity for Tomato spotted wilt virus (TSWV) in the SunOleic 97R × NC94022 Recombinant Inbred Line (RIL) population

Year	Range	Mean	Variance	Standard deviation	Skewness	Kurtosis	w-test^a^
2010	1–5	2.4207	1.1568	1.0755	1.0787	1.1685	0.9053
2011	1–5	2.41	0.85	0.92	0.80	0.30	0.92
2013	1.17–5	2.85	0.94	0.97	0.52	−0.29	0.95
2019	0–5	1.94	0.78	0.88	0.22	−0.20	0.97
2020	0–5	2.65	0.75	0.87	0.04	−0.64	0.96

^a^Shapiro–Wilk statistic test

### Linkage mapping and QTL analysis using ‘Axiom_*Arachis*-v2’ SNP array

The parental lines and the whole “S” population of 352 RILs were genotyped using the 48K ‘Axiom_*Arachis*-v2’ SNP array [16]. There were 5,706 polymorphic SNPs between the two parents. Among these, 5,035 (88.24%) were homozygous (AA) in SunOleic 97R, and 5,125 (89.82%) were homozygous (BB) in NC94022. Notably, 4,457 (78.06%) SNPs were homozygous in both parental lines. The polymorphic SNPs were distributed across all 20 peanut chromosomes, with chromosome A01 containing the highest number (581 SNPs).

A high-resolution linkage map was constructed after filtering out SNPs with severe segregation distortion (*P* < 0.001), more than 10% missing data, or those that were unlinked. The linkage map published recently for oil trait study [18] has been used for this study. Briefly, the final map had 3,141 SNPs in 20 linkage groups corresponding to the 20 cultivated peanut chromosomes and covered a total length of 3,051.8 cM. The density of this high-resolution map was 0.97 cM per loci, with sub-genomes A and B covering 1,425.33 cM and 1,626.49 cM, respectively. Chromosome A01 had the highest density, with 367 markers and an average distance of 0.17 cM between loci [18].

**Fig. 3:**
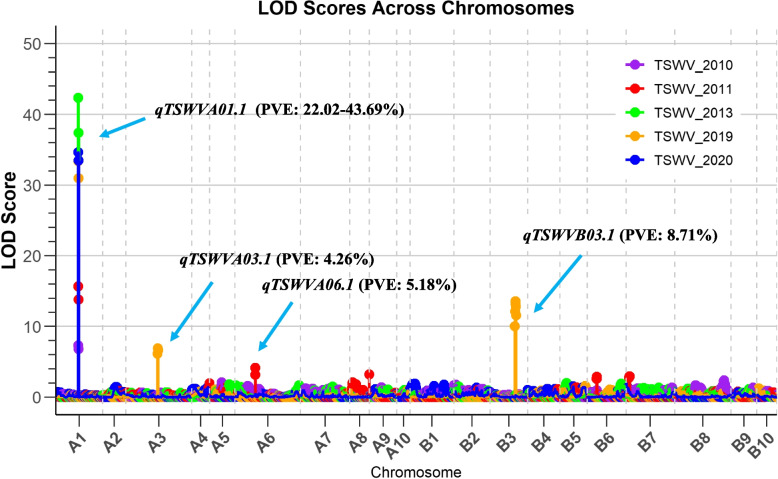
Mapping of QTLs for Tomato spotted wilt virus (TSWV) resistance using version 2 peanut SNP array. The x-axis represents the twenty linkage groups corresponding to the 20 chromosomes of cultivated peanut. The y-axis shows the LOD (Logarithm of the Odds) scores. Different lines indicate different years. Dashed vertical lines are borders of the chromosomes

**Fig. 4:**
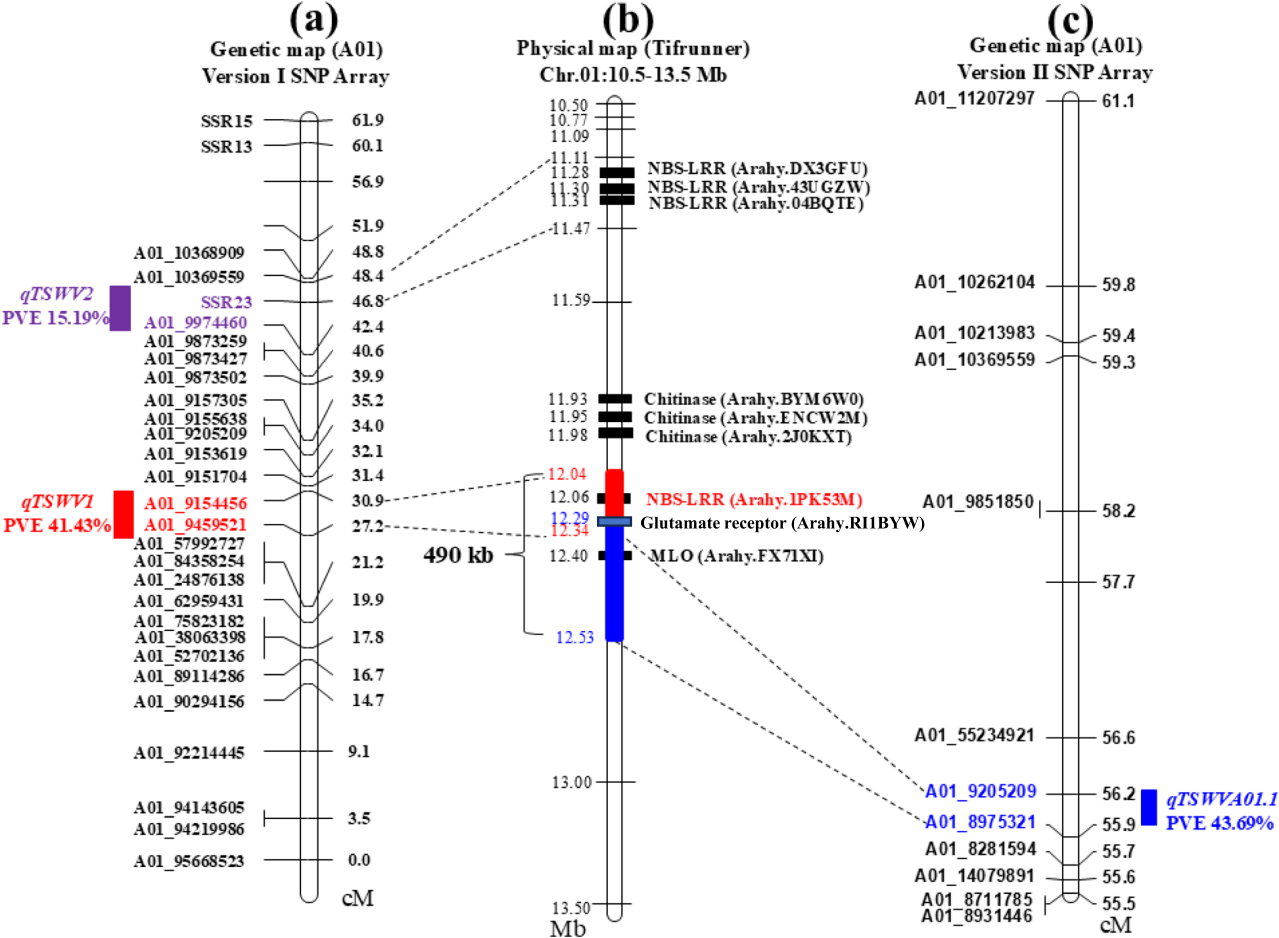
Integrated analysis of genetic map and physical map to identify the potential intervals harboring the candidate genes conferring the resistance to Tomato spotted wilt virus (TSWV). **a** linkage map of A01 constructed using version 1 peanut SNP Array and SSRs. **b** a partial physical map of A01 of community reference genome of cultivated peanut Tifrunner with candidate genes. **c** a partial linkage map of A01 constructed using version 2 peanut SNP array

Four QTLs associated with TSWV resistance were identified (Fig. 3 and Table 2). The identified QTLs had logarithm of odds (LOD) scores ranging from 4.14 to 42.34 and collectively explained between 4.26% and 43.69% of observed phenotypic variation (PVE). The genetic intervals of these QTLs varied from as narrow as 0.28 cM (*qTSWVA01.1*) to as wide as 2.7 cM (*qTSWVB03.1*). The major QTL on chromosome A01, *qTSWVA01.1*, had 43.69% of PVE. The QTL was consistently detected across all five years of data, highlighting its robustness and reliability. Furthermore, this locus was mapped to a very small genetic interval, 0.28 cM, making it a prime candidate for further study to identify the underlying genes responsible for TSWV resistance (Table 2 and Fig. 4).

**Table 2:** QTL for resistance to Tomato spotted wilt virus (TSWV) in peanut across five years phenotypes using version 2 peanut SNP array

QTL	LG^a^	Year	Flanking marker	Genetic interval	Length (cM)	ADD^b^	LOD^c^	PVE^d^ (%)
*qTSWVA01.1*	A01	2013	A01_8975321- A01_9205209	55.938- 56.219	0.281	0.638	42.3389	43.6891
		2019				0.5116	33.3796	24.7461
		2011				0.4183	15.678	22.0141
		2010				0.5723	7.3222	27.9784
		2020				0.5299	34.6767	37.7683
*qTSWVB03.1*	B03	2019	B03_34461271-B03_32707264	112.839–115.539	2.7	0.3035	13.5764	8.7071
*qTSWVA06.1*	A06	2011	A06_14820727-A06_14440957	89.709–90.044	0.335	0.2016	4.1439	5.1845
*qTSWVA03.1*	A03	2019	A03_39367411-A03_35623960	139.731–141.27	1.539	−0.2115	6.888	4.256

^a^linkage group

^b^additive effect value

^c^logarithm of odds score

^d^phenotypic variance explained by individual QTL

### QTL analysis using ‘Axiom_*Arachis*-v1’ SNP array and SSR marker

As a supplemental study and comparison with ‘Axiom_*Arachis*-v2’ SNP array, we genotyped a subset of 143 randomly selected RILs from the “S” population using the 58K ‘Axiom_*Arachis*_v1*’* SNP array [11, 12]. There were 1,741 polymorphic SNPs between the two parents. Among these polymorphic SNPs, 1,309 (75.2%) were homozygous (AA) in SunOleic 97R, and 1,276 (73.2%) were homozygous (BB) in NC94022 (Figure S2). The polymorphic SNPs were distributed across the A and B sub-genomes, with 929 SNPs in the A sub-genome and 812 in the B sub-genome. Chromosome A06 had the highest number of SNPs (163), and chromosomes A10 and B10 had the fewest SNPs (37 each) (Figure S2). A genetic linkage map was also constructed, resulting in 20 linkage groups spanning a total length of 3,380.95 cM and containing 1,012 markers (Table S1), which has 2,129 fewer markers than the map as reported using the SNP array version 2 [18]. The average distance between markers across the entire map was 3.34 cM. Linkage group A01 included 31 markers, with an average marker distance of 1.99 cM. Two QTLs associated with resistance to TSWV were identified, both located in linkage group A01 (Table S2, Fig. 4). The LOD scores for these QTLs were 4.52 and 16.72, accounting for 15.19% and 41.43% PVE, respectively. The QTL named *qTSWV*1, located between markers A01_9459521 and A01_9154456 (3.6 cM), explained the highest PVE of 41.43% (Table S2, Fig. 4).

### TSWV resistance locus *PSWDR-*1 resides in a recombination “cold spot” region on chromosome A01

We previously mapped a major TSWV resistance QTL on chromosome A01 at about 10 Mb on a physical map (Figure S1) [4, 17]. To fine map the resistance locus, we used the peanut SNP arrays to genotype the entire population of 352 RILs. The major QTL from SNP array version 1 (*qTSWV*1) possessed a PVE of 41.43% and was mapped to a 295 kb interval on the community reference chromosome A01 (Chr.01: 12.04–12.34 Mb) (Fig. 4). The major QTL identified using the version 2 SNP array (*qTSWVA01.1*) with a PVE of 43.69% was localized to a 235 kb physical interval on the community reference chromosome A01 (Chr.01: 12.29–12.53 Mb) (Fig. 4). Notably, the two major QTLs identified from both SNP arrays overlapped to make a combined region of 0.49 Mb on chromosome A01 (Chr.01: 12.04 –12.53 Mb) (Fig. 4). We named this combined region as *PSWDR-*1, a peanut spotted wilt disease resistance locus to TSWV.

**Fig. 5:**
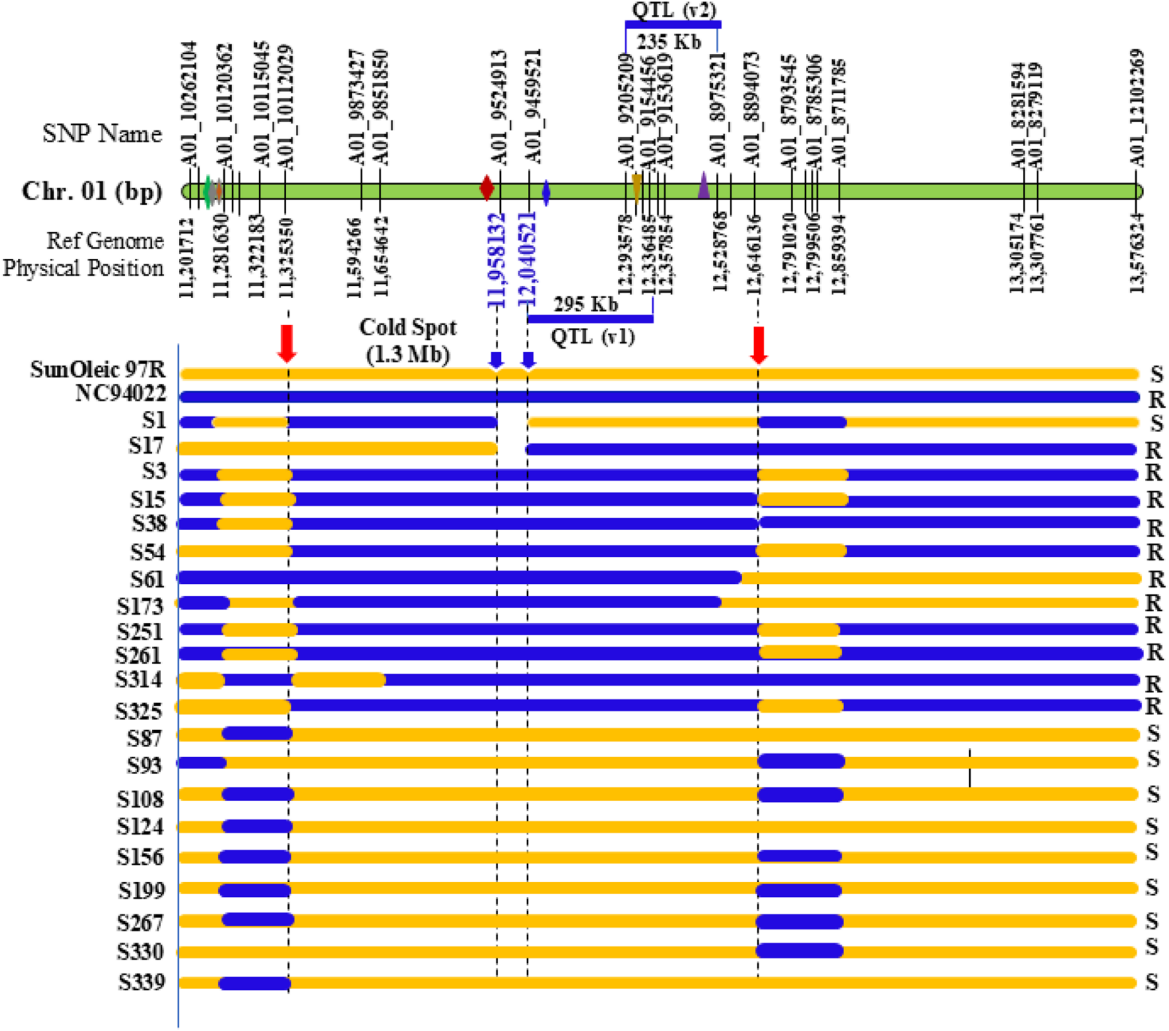
Fine-mapping of Tomato spotted wilt virus (TSWV) resistance QTL on chromosome A01. The green bar represents the physical map of chromosome A01, with SNPs labeled above and physical locations below. QTLs identified using Version-I and Version-II SNP arrays are shown as blue bars above and below the chromosome, with QTL (v1) spanning a 295 Kb region and QTL (v2) spanning a 235 Kb region. Gene representations include the red diamond (Chitinase family protein, Arahy.ENCW2M), the blue diamond (candidate NBS-LRR gene, Arahy.1PK53M), the yellow inverted triangle (candidate glutamate receptor gene, Arahy.RI1BYW), the purple triangle (candidate MOL defense protein, Arahy.FX71XI), and clustered diamonds (three additional NBS-LRR genes). Parental lines (SunOleic97R and NC94022), recombinant inbred lines (RIL-S1 and RIL-S17), along with 10 resistant and 9 susceptible RILs, are displayed below the green bar. Blue regions represent chromosome segments from NC94022, while orange regions represent segments from SunOleic97R. A recombination cold spot near the QTL region is highlighted with red arrows

The independent recombinant genotypes were compared in the combined region of *PSWDR-*1 and positioned on reference genome with physical positions (Fig. 5). Interestingly, a 1.3 Mb recombination "cold spot" was identified between 11.325 Mb and 12.646 Mb on chromosome A01, characterized by a significantly reduced recombination frequency across the RIL population based on both SNP arrays genotype data. Only two recombination events, RIL-S1 and RIL-S17, in the entire population of 352 RILs were detected within this region at about 12 Mb and both lines exhibited contrasting phenotypes (Figure S3). The RIL-S17 was resistant to TSWV and RIL-S1 was susceptible through the years. These two lines further were sequenced at 20X coverage and aligned to the physical map. This confirmed the low recombination rate in this region of “cold spot” by revealing only five SNPs within the interval (Table 3).

**Table 3:** List of SNPs identified in the QTL region on chromosome A01 and their significance

Position	Ref	Alt	Gene ID	Genomic region	Gene description	S1	S17	Amino acid change	SNP effect
12,060,020	T	C	-	Intergenic	-	C	T		
12,064,257	T	C	1PK53M	Intronic	Disease resistance protein (TIR-NBS-LRR class)	**T**	**C**		
12,064,414	G	A	1PK53M	Intronic	Disease resistance protein (TIR-NBS-LRR class)	**G**	**A**		
12,205,766	G	A	-	Intergenic	-	A	G		
12,309,043	T	C	VKPB3K	Exonic	Putative nuclease HARBI1-like	C	T	T14A	Non-synonymous

These recombination events enabled the fine-mapping of the QTL region to a 489-kb interval between 12.04 Mb and 12.529 Mb by comparing the resistant segments (colored in blue) and susceptible segments (colored in orange) in the resistant and susceptible RILs along with parents (Fig. 5). This refined interval now represents the most promising candidate region for genes conferring resistance to TSWV. This region was named as *PSWDR-*1, a peanut spotted wilt disease resistance locus to TSWV.

To identify candidate genes potentially responsible for TSWV resistance, the 489-kb candidate region between 12.04 Mb and 12.529 Mb (flanked by markers A01_9524913 and A01_8975321) and the adjacent regions was explored in the reference genome. There are 42 genes within this interval (Table S3). Based on the available transcriptome data for tetraploid peanuts [19], there were 17 genes with no expression in leaves, lateral stems, main stems, or seedlings were excluded. The remaining 25 genes were considered potential candidates associated with *PSWDR-*1. Among these, the particularly noteworthy genes were NBS-LRR gene (*Arahy.1PK53M*), glutamate receptor (*Arahy.RI1BYW*), and MLO-like protein 8 gene (*Arahy.FX71XI*) (Fig. 5, Table S4).

## Discussion

### SNP array-based high-density linkage mapping and QTL identification

Tomato spotted wilt virus (TSWV) is a major pathogen impacting peanut production, particularly in the southeastern U.S. To breed resistant cultivars, identifying QTLs and genes associated with TSWV resistance and utilizing marker assisted selection (MAS) are crucial. The quality of a genetic linkage map is essential for accurate QTL mapping and depends on factors such as the number of markers, marker density, and population size. Therefore, we used the newly improved peanut SNP array version 2 [16] to genotype the whole “S” population of 352 RILs. A high-density genetic linkage map was constructed as reported by Wang et al. [18] with 3,141 SNP markers, 30,51 cM in length, and the marker density of 0.17 cM on chromosome A01. We also used version 1 of the peanut SNP array [11, 12] as a supplemental study and comparison with the version 2 SNP array. As a result, the linkage map had 1,012 markers and was 3,380.95 cM in length. The average distance between markers across the entire map was much lower than the version 2 (3.34 cM vs. 0.97 cM, respectively) and the markers density on chromosome A01 were much lower using version 1 than version 2 SNP array (1.99 cM vs. 0.17 cM, respectively). This supports the improvement in genotyping that the version 2 SNP array has over the version 1 SNP array, facilitating higher resolution genetic mapping of traits.

The results of the genetic mapping and QTL studies revealed the long-sought-after peanut spotted wilt disease resistance locus *PSWDR*−1 on chromosome A01 (Figure S1) and that this locus is located in a recombination cold-spot. Comparisons of the physical sequences of this locus between two rare recombinants in this cold spot, RIL-S1 and RIL-S17, confirms the recombination cold spot (Figure S3). Within *PSWRD*−1 there are three potential candidate genes, an NBS-LRR disease resistance gene, a glutamate receptor, and an MLO-like protein.

In recent years, several high-density genetic maps (HDGMs) for peanut have been developed using sequencing-based methods, including specific length amplified fragment sequencing (SLAF-seq) [20–22] double-digest restriction-site-associated DNA sequencing (ddRAD-seq) [23], and whole-genome resequencing (WGRS) [24]. This study presents the first SNP Array-based HDGM for TSWV resistance in cultivated peanut, surpassing the number of loci and density of most previously reported HDGMs. Unlike other sequencing-based methods, the SNP array approach avoids issues related to consistent regional sequencing depth throughout the population and biases from restriction enzymes. The markers included in the peanut SNP array are pre-screened and evenly distributed across chromosomes, with many specific to the A and B sub-genomes [11, 16].

Since cultivated peanuts are tetraploids with two highly similar sub-genomes, sub-genome-specific markers are essential for accurate QTL mapping. In this study, over 78% of the markers from the version 2 SNP Array were specific to the A and B sub-genomes, slightly exceeding the 74% reported by Clevenger et al. [16]. Overall, SNP arrays offer an efficient and reliable approach for constructing genetic maps and identifying QTLs in peanuts.

It is worth to note that the “S” population has been used for other trait mapping studies, including oil content, and resistance to leaf spot. Previous studies have identified several QTLs associated with these traits using earlier linkage maps [25, 26]. The high-density genetic map developed in this study provides an opportunity to further refine and re-map these QTLs, contributing to future breeding efforts.

### Enhancing TSWV QTL mapping precision in peanut with a larger population, high-density SNP array, and multi-year phenotyping

The construction of a linkage map depends on the frequency of recombination between markers during crossover events in homologous chromosomes. The mapping population size plays a critical role in determining the map's quality and accuracy. In this study, we utilized 352 RILs of the “S” population to develop a high-density linkage map. This population size is significantly larger than those used in previous studies for constructing HDGMs in peanut [21–24].

Using a larger mapping population provides several critical advantages. A larger population captures more recombination events, to reduce the genetic distance between markers, thus facilitating more accurate QTL localization [27]. This further narrows confidence intervals around QTL positions, enabling finer-scale mapping [28]. A larger sample size can mitigate the impact of segregation distortion, ensuring more reliable and robust linkage analysis [29]. Although previous studies identified TSWV-related QTLs on chromosome A01, the reported intervals and specific locations varied substantially (Figure S1) [4–7, 17]. The major limitations in these earlier studies were the smaller population sizes and the types and sources of molecular markers used. In contrast, this study’s substantially larger population and high-quality SNP array were pivotal in refining the QTL region associated with TSWV resistance. The increased population size enabled the detection of rare recombination events, such as those identified in lines S1 and S17 within the recombination cold spot, significantly improving the resolution of the QTL region.

Another crucial factor influencing QTL mapping quality is the accuracy and reproducibility of phenotyping data. Phenotypic data collected across multiple years can yield variable QTL results. However, the major QTL (*qTSWVA01.1*) identified using the version 2 SNP array consistently appeared across all five years of phenotypic data, underscoring the reliability and stability of the identified QTL.

In summary, the integration of a larger mapping population, high-density and quality markers, and multi-year phenotyping data is essential for fine-scale mapping of QTLs across diverse crops and traits. Together, these factors enhance the precision, reliability, and robustness of QTL mapping.

### Candidate genes within the resistance locus *PSWDR*−1 and the recombination “cold spot”

Nucleotide-binding site leucine-rich repeat (NBS-LRR) genes constitute one of the largest families of disease resistance (R) genes in plants [30, 31]. Most R genes identified through map-based cloning belong to this family. Notable examples include rice blast resistance genes *Pi9* and *Pi36* [32, 33], the leaf rust resistance gene *Lr10*, and wheat powdery mildew resistance genes *Pm3*, *Pm8*, and *Pm21* [34–37]. In tomatoes, the *Sw5* genes, which confer resistance to Tomato spotted wilt virus (TSWV), also encodes NBS-LRR proteins [38].

In this study, one NBS-LRR gene (*Arahy.1PK53M.1*) was identified within the QTL region and was expressed in peanut leaves. A BLAST search against the SwissProt database revealed homologs from species including *Nicotiana benthamiana*, *N. glutinosa*, *Vitis rotundifolia*, *Arabidopsis thaliana*, and *Linum usitatissimum*, with alignment scores greater than 300. Phylogenetic analysis showed that *Arahy.1PK53M.1* aligns closely with the L6 resistance protein from flax (*L. usitatissimum*), a TIR-NBS-LRR receptor, but is distant from tomato’s *Sw5b* protein (Figures S4A and S4B). The L6 protein in flax triggers immune responses by recognizing effectors from *Melampsora lini*, the rust fungus [39, 40]. An InterPro domain search identified *Arahy.1PK53M.1* as a TIR-NBS-LRR-type receptor, whereas *Sw5b* belongs to the CC-NBS-LRR-type family [41] (Figures S4C, S4D). The difference between these receptor types could highlight distinct mechanisms of action. NBS-LRR type R genes play an important role in the plant’s immune response and can confer host resistance to bacteria, fungi, oomycetes, viruses, and even sucking insects, which are crucial vectors for viral diseases. In peanuts, NBS-LRR proteins can potentially detect the presence of TSWV or its insect vectors and activate a series of immune responses, including the production of reactive oxygen species (ROS) and the expression of other defense-related genes to defend against TSWV. Further functional investigation is required to confirm whether *Arahy.1PK53M.1* confers resistance specifically to TSWV or other pathogens.

The *PSWDR*−1 locus also contained an MLO protein (*Arahy.FX71XI*), a known susceptibility gene in many crops. The peanut MLO protein shares 48.36% identity and 62% similarity with the barley MLO protein (Figure S5). Loss-of-function mutations in barley MLO result in resistance to the powdery mildew fungus *Blumeria graminis f. sp. hordei* [42, 43]. Similarly, transposable element insertion in cucumber *CsaMLO8* confers resistance to powdery mildew [44]. Even though MLO-like proteins were initially discovered for their role in plant-powdery mildew interactions, evidence suggests they are also involved in regulating drought stress responses [45] and biotic stress responses to bacterial and oomycete pathogens [46]. Given the conservation of MLO proteins and their known roles in broader pathogen interactions, peanut *Arahy.FX71XI* could similarly mediate resistance or susceptibility to TSWV or other diseases.

The third gene identified in this region is *Arahy.RI1BYW.1*, which encodes a glutamate receptor-like (GLR) protein. This protein shares 58% identity and 76% similarity with GLR3.6 from *A. thaliana*. GLRs are thought to regulate long-distance defense signaling in response to herbivory and wounding through ion channels [47, 48]. In cotton, the homologous gene *GhGLR4.8* plays a role in resistance to *Fusarium* wilt, and GLRs in oilseed rape are involved in defense against *Sclerotinia sclerotiorum*, the white mold fungus [49, 50]. Although the involvement of GLRs in viral defense has not yet been reported, their known roles in calcium-based long-distance defense signaling in response to herbivory and wounding suggest that peanut GLR genes could confer resistance to TSWV by activating defense mechanisms in response to the wounding caused by disease-transmitting insects. Therefore, they are relevant candidates for further investigation.

In summary, the NBS-LRR, MLO, and GLR genes identified within peanut spotted wilt disease resistance locus *PSWDR*−1 are promising candidates for conferring resistance to TSWV. Further studies, such as transcriptome analyses and functional assays, are needed to confirm their roles and determine the specific mechanisms underlying their contributions to TSWV resistance. Investigating the functions of these candidate genes could also provide new insights into cross-resistance against other pathogens and offer valuable tools for breeding resistant peanut cultivars.Interestingly, these candidate genes are located in a recombination “cold-spot”, which is crucial for genetic studies and breeding. These regions ensure that favorable allele combinations are inherited together, enhancing plant fitness. They also facilitate accurate QTL mapping, as indicated in our study, allowing precise identification of traits of interest. These regions act as reservoirs of genetic diversity, supporting long-term adaptability and evolutionary processes. Understanding recombination cold-spots is essential for advancing plant breeding and developing superior crop varieties.

## Conclusion

This study successfully mapped two major QTLs associated with TSWV resistance in cultivated peanut, located on chromosome A01, using peanut SNP array version 1 and version 2. The identified QTLs, *qTSWV1* and *qTSWVA01.1*, respectively, each explained a significant proportion of the phenotypic variance across multiple years. By integrating physical map and linkage map, we confirmed a recombination “cold spot” hosting the peanut spotted wilt disease resistance locus *PSWDR*−1. This locus was refined to a 489-kb interval between 12.04 Mb and 12.529 Mb. This refinement was made possible by analyzing the detailed genotyping and recombination events in this region and only two lines, RIL-S1 and RIL-S17 with contrasting phenotypes, were crossed over within the “cold spot” on chromosome A01. Further investigation of the refined interval revealed 25 potential candidate genes, among which one NBS-LRR gene (*Arahy.1PK53M*), a glutamate receptor (*Arahy.RI1BYW*), and an MLO-like protein 8 gene (*Arahy.FX71XI*) are promising candidates for conferring TSWV resistance. These findings provide a foundation for future functional studies aimed at validating the roles of these candidate genes in resistance, advancing the development of TSWV-resistant peanut varieties.

## Materials and methods

### Mapping population, phenotyping, and DNA extraction

A mapping population of 352 recombinant inbred lines (RILs) from a cross between SunOleic 97R (susceptible to TSWV) and NC94022 (highly resistant to TSWV) [4, 5, 17], referred to as the “S” population, were used to construct a genetic linkage map and perform QTL mapping to identify loci associated with TSWV resistance. This population has been used in several previous studies [4, 5, 17]. The female parent, NC94022, is highly resistant to TSWV and was derived from a cross between N91026E (moderately susceptible to TSWV) and SSD6 (PI 576638), a highly resistant line [51]. The male parent, SunOleic 97R, is known for its susceptibility to TSWV (Fig. 1).

The parents and the entire population of the “S” population were grown in 2013, 2019, and 2020, while a subset was grown in 2010, 2011, at the Bellflower Farm in Tifton, GA, USA. A randomized completed block design with three replications was used. The experimental plots were two-row plots (1.5 m long with 0.9 m row spacing) and a seeding rate of 10 seeds per meter. TSWV disease intensity was evaluated using an improved 0–5 scale, where 0 indicates no symptoms and 5 indicates severe symptoms, following established methods [52]. The phenotype data have been provided in supplementary Table S6.

Leaf samples from both parents and RILs were collected for DNA extraction using the GeneJET Genomic DNA Purification Kit (Thermo Fisher Scientific, Waltham, MA USA). The quality and concentration of DNA samples were assessed using a NanoDrop ND-1000 spectrophotometer (NanoDrop Tech, Wilmington, DE, USA), ensuring high-quality DNA for subsequent analyses.

Statistical analyses of the phenotypic data including Shapiro–Wilk test (w-test) [17] were also generated by the QTL IciMapping V4.1 (Table 1). The Shapiro–Wilk (w) test indicated that the distribution of the phenotypic data across years were normal in most.

### Genotyping using SNP arrays

The entire “S” population as reported by Qin et al. [4] were genotyped using the both peanut SNP Array version 1 (58K ‘Axiom_*Arachis*_v1) [11, 12] and version 2 (‘Axiom_*Arachis’* 48 K) [16]. DNA samples were submitted to Affymetrix following the workflows described in our previous studies [13]. Briefly, the raw genotyping data obtained from Affymetrix were stored in.CEL files and analyzed using the Axiom Analysis Suite (Thermo Fisher Scientific, Waltham, MA, USA) for quality control and selecting high-quality SNPs. The polymorphic SNPs identified between the parental lines were then selected for QTL mapping to identify loci associated with key traits. To assess the distribution and density of SNP markers across chromosomes [18], the data were visualized using the R package CMplot [53], which aids in identifying regions with high SNP density. The genotype data have been provided for both SNP arrays in supplementary Table S7 and Table S8, respectively.

### Linkage map construction and QTL analysis

The linkage map was constructed using JoinMap 5.0 software (Kyazma), with a Chi-squared (χ^2^) test applied to exclude markers exhibiting distorted segregation (*P* > 0.001). The Kosambi mapping function was used to convert recombination ratios into genetic distances (centimorgans, cM), and all linkage groups were calculated at a minimum LOD score of 5. The resulting linkage maps were drawn using MapChart 2.3 software [54].

For QTL analysis, inclusive composite interval mapping of additive effects (ICIM-ADD) was conducted using QTL IciMapping V4.1 [55]. The LOD threshold for identifying putative QTLs was set at 2.5, determined by 1,000 permutations at P < 0.05. According to standard criteria, QTLs with PVE > 10% were classified as major QTLs, while those with lower PVE were considered minor QTLs [21, 56]. This approach helps to reliably identify significant genomic regions associated with target traits.

### Physical mapping and prediction of candidate genes

To identify candidate genes associated with the target trait, the physical positions of SNP markers were determined by aligning the sequences of SNP probes with the Tifrunner reference genome sequences [9] using a BLAST search [57]. The BLAST search was conducted with stringent criteria of E-value < 10^^−5^ and a minimum similarity of > 95% between the query and database sequences. If multiple locations were identified for the same SNP probe, only the best match was considered. An integrated analysis of the genetic and physical maps was performed by positioning the SNP markers on their respective chromosomes, ensuring consistency between genetic distances and physical locations. The functions of potential candidate genes were further annotated using BlastX against the Nr (nonredundant) database [58], providing insights into their possible roles in relevant biological processes and pathways.

### Library construction and sequencing

To refine the physical map of the TSWV resistance QTL region on chromosome A01 and identify potential candidate genes, a whole-genome shotgun sequencing strategy was employed for two parent lines and two recombinant inbred lines (RILs), S1 (susceptible) and S17 (resistant). High-depth sequencing (20X coverage) was performed using paired-end sequencing libraries with an insert size of 300 bp on an Illumina HiSeq 2000 platform (Illumina, San Diego, CA, USA) at the HudsonAlpha Institute for Biotechnology (Huntsville, AL, USA). For each line, paired reads of 150 bp in length were generated. Filtered reads were aligned to version 2 of the *Arachis hypogaea* cv. Tifrunner reference genome assembly available at PeanutBase [57, 59]. The alignment files obtained from this mapping were used to identify polymorphic haplotypes, following the method described by Agarwal et al. [17]. Single Nucleotide Polymorphisms (SNPs) were further annotated using Annovar [60], providing insights into their functional impact and potential roles in the resistance mechanism.

## Supplementary Information

Supplementary Material 1.

Supplementary Material 2.

Supplementary Material 3.

## Data Availability

The phenotype and genotype data are provided within the manuscript or supplementary information files and the raw sequencing data of S1 and S17 are available in the NCBI Sequence Read Archive under accession number BioProject: PRJNA1196376 (https://www.ncbi.nlm.nih.gov/bioproject/PRJNA1196376). The parental lines’ sequencing data used in this study are available from the corresponding author upon request (which will be published in another manuscript).
