# Supplementary material for: High-resolution genetic and physical mapping reveals a peanut spotted wilt disease resistance locus, PSWDR-1, to Tomato spotted wilt virus (TSWV), within a recombination cold-spot on chromosome A01
Source: BMC Genomics. 2025 Mar 6;26:224. doi: 10.1186/s12864-025-11366-7 (PMC11887336; doi:10.1186/s12864-025-11366-7)
Supplement: Supplementary file 1 — Supplementary Material 1. [file 12864_2025_11366_MOESM1_ESM.pptx]

## Slide 1
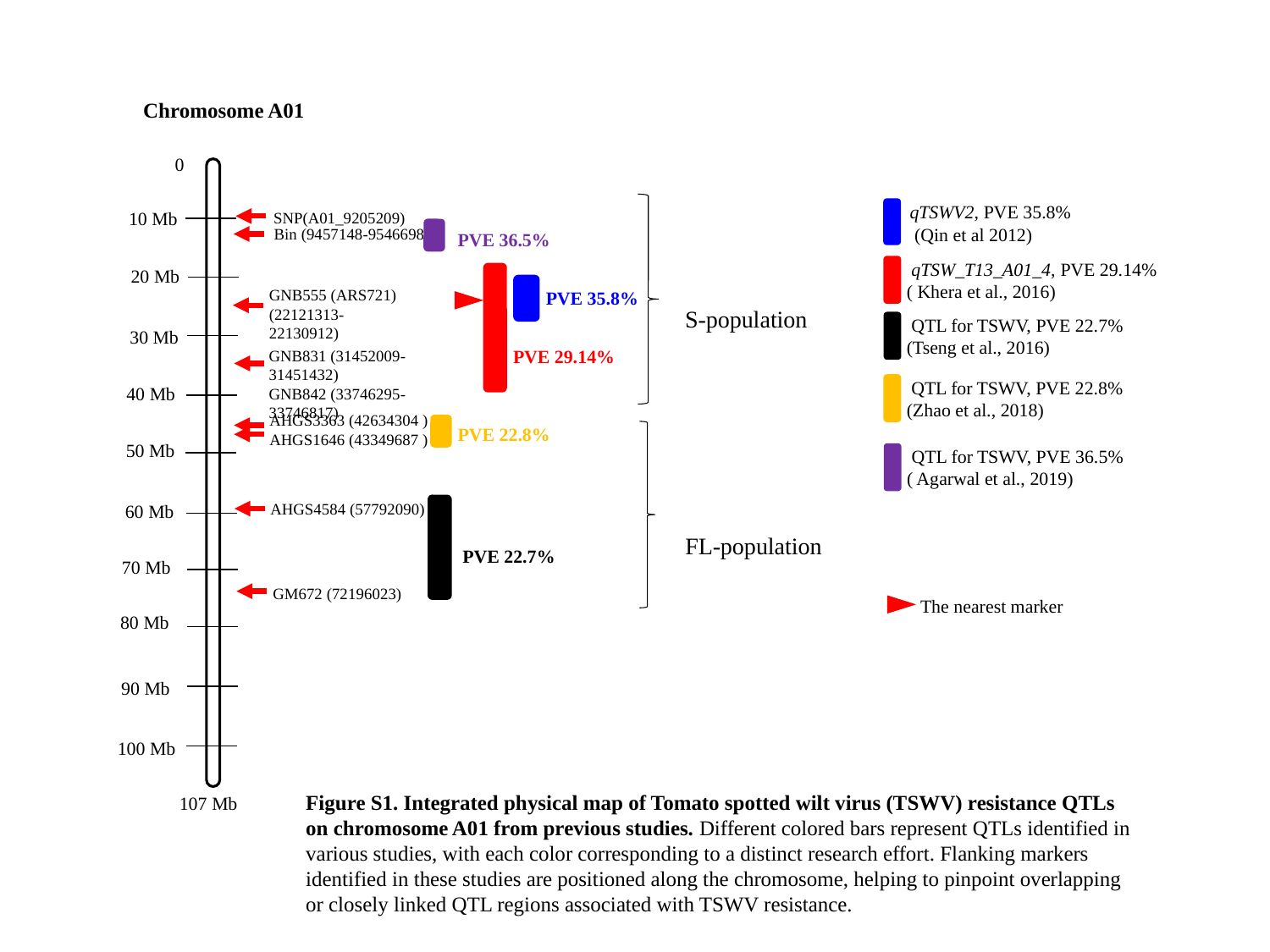

Chromosome A01
0
10 Mb
20 Mb
30 Mb
40 Mb
50 Mb
60 Mb
70 Mb
80 Mb
90 Mb
100 Mb
107 Mb
qTSWV2, PVE 35.8%
 (Qin et al 2012)
SNP(A01_9205209)
Bin (9457148-9546698)
PVE 36.5%
 qTSW_T13_A01_4, PVE 29.14%
( Khera et al., 2016)
GNB555 (ARS721)
(22121313-22130912)
PVE 35.8%
S-population
 QTL for TSWV, PVE 22.7%
(Tseng et al., 2016)
PVE 29.14%
GNB831 (31452009-31451432)
GNB842 (33746295-33746817)
 QTL for TSWV, PVE 22.8%
(Zhao et al., 2018)
AHGS3363 (42634304 )
AHGS1646 (43349687 )
PVE 22.8%
 QTL for TSWV, PVE 36.5%
( Agarwal et al., 2019)
AHGS4584 (57792090)
FL-population
PVE 22.7%
GM672 (72196023)
 The nearest marker
Figure S1. Integrated physical map of Tomato spotted wilt virus (TSWV) resistance QTLs on chromosome A01 from previous studies. Different colored bars represent QTLs identified in various studies, with each color corresponding to a distinct research effort. Flanking markers identified in these studies are positioned along the chromosome, helping to pinpoint overlapping or closely linked QTL regions associated with TSWV resistance.

## Slide 2
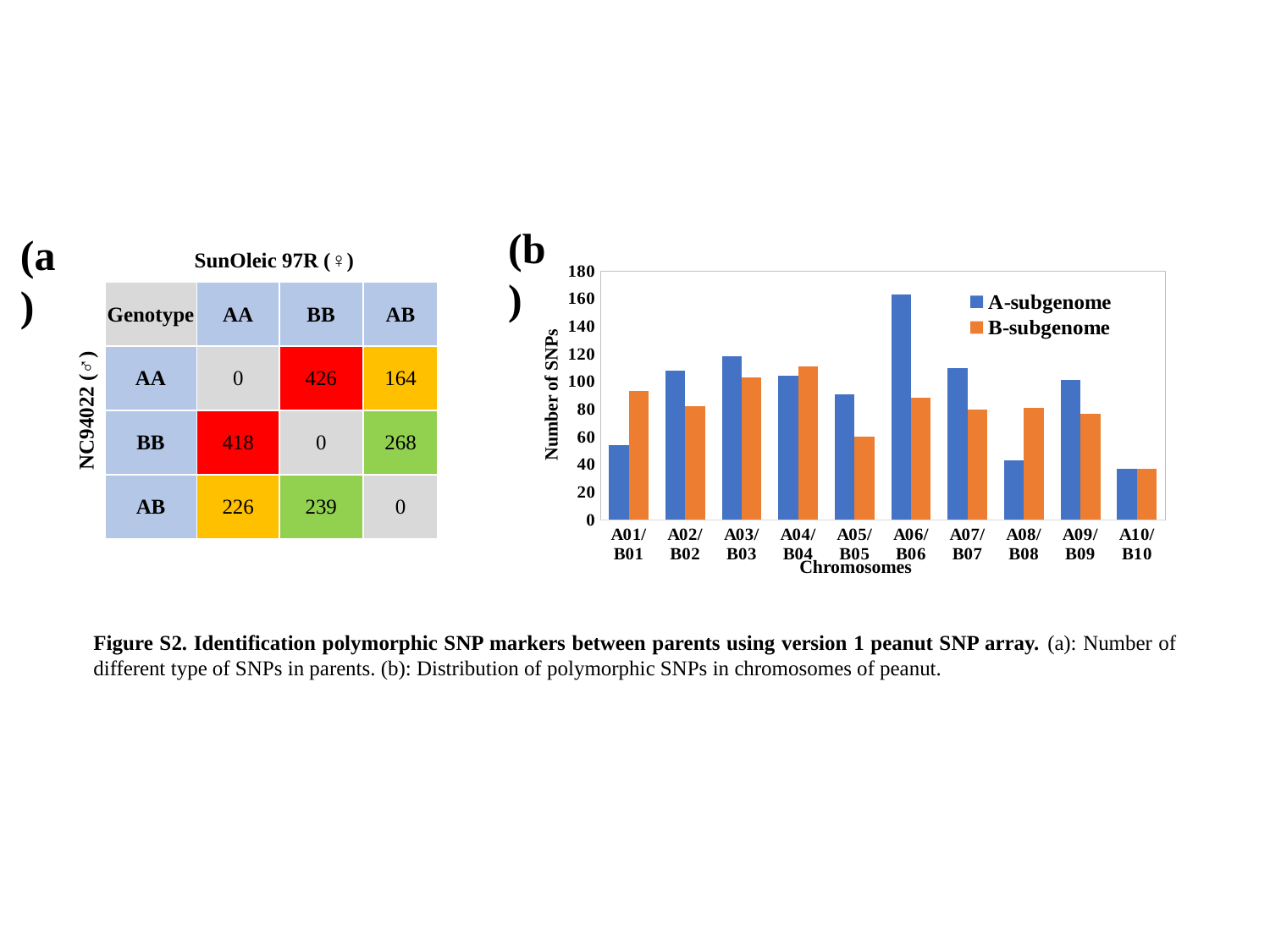

(b)
(a)
SunOleic 97R (♀)
### Chart
| Category | A-subgenome | B-subgenome |
|---|---|---|
| A01/B01 | 54.0 | 93.0 |
| A02/B02 | 108.0 | 82.0 |
| A03/B03 | 118.0 | 103.0 |
| A04/B04 | 104.0 | 111.0 |
| A05/B05 | 91.0 | 60.0 |
| A06/B06 | 163.0 | 88.0 |
| A07/B07 | 110.0 | 80.0 |
| A08/B08 | 43.0 | 81.0 |
| A09/B09 | 101.0 | 77.0 |
| A10/B10 | 37.0 | 37.0 || Genotype | AA | BB | AB |
| --- | --- | --- | --- |
| AA | 0 | 426 | 164 |
| BB | 418 | 0 | 268 |
| AB | 226 | 239 | 0 |
Number of SNPs
NC94022 (♂)
Chromosomes
Figure S2. Identification polymorphic SNP markers between parents using version 1 peanut SNP array. (a): Number of different type of SNPs in parents. (b): Distribution of polymorphic SNPs in chromosomes of peanut.

## Slide 3
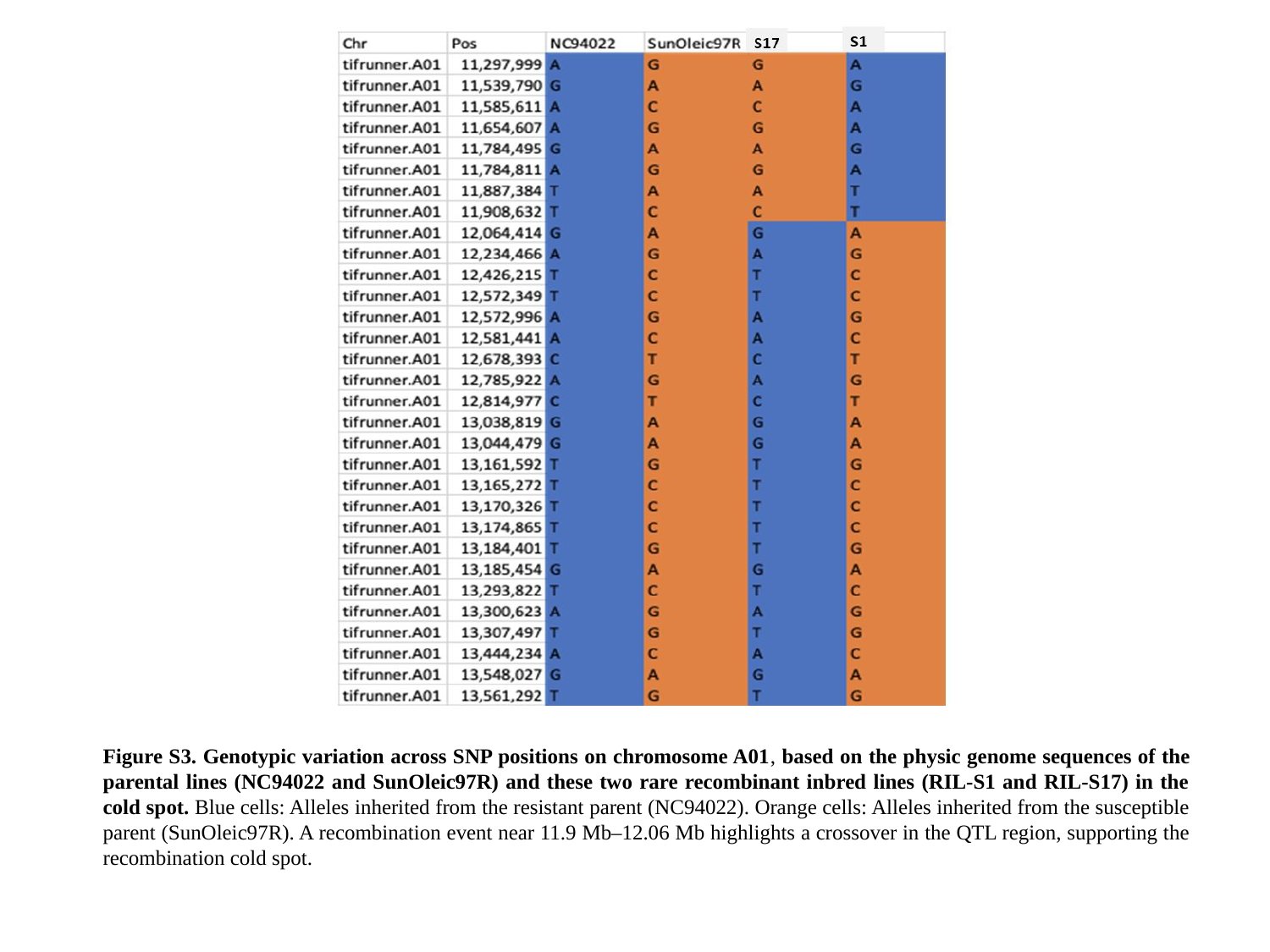

Figure S3. Genotypic variation across SNP positions on chromosome A01, based on the physic genome sequences of the parental lines (NC94022 and SunOleic97R) and these two rare recombinant inbred lines (RIL-S1 and RIL-S17) in the cold spot. Blue cells: Alleles inherited from the resistant parent (NC94022). Orange cells: Alleles inherited from the susceptible parent (SunOleic97R). A recombination event near 11.9 Mb–12.06 Mb highlights a crossover in the QTL region, supporting the recombination cold spot.

## Slide 4
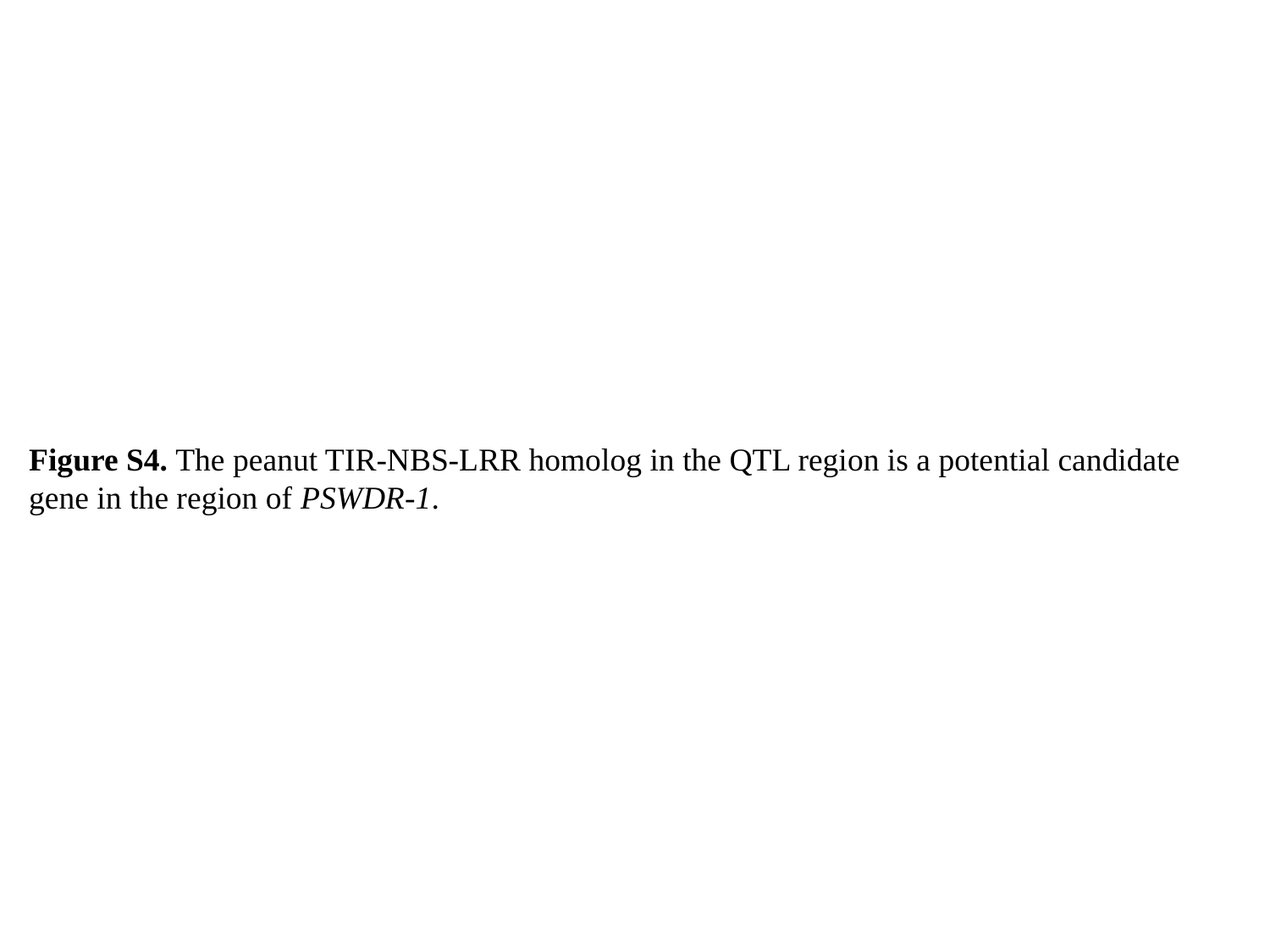

Figure S4. The peanut TIR-NBS-LRR homolog in the QTL region is a potential candidate gene in the region of PSWDR-1.

## Slide 5
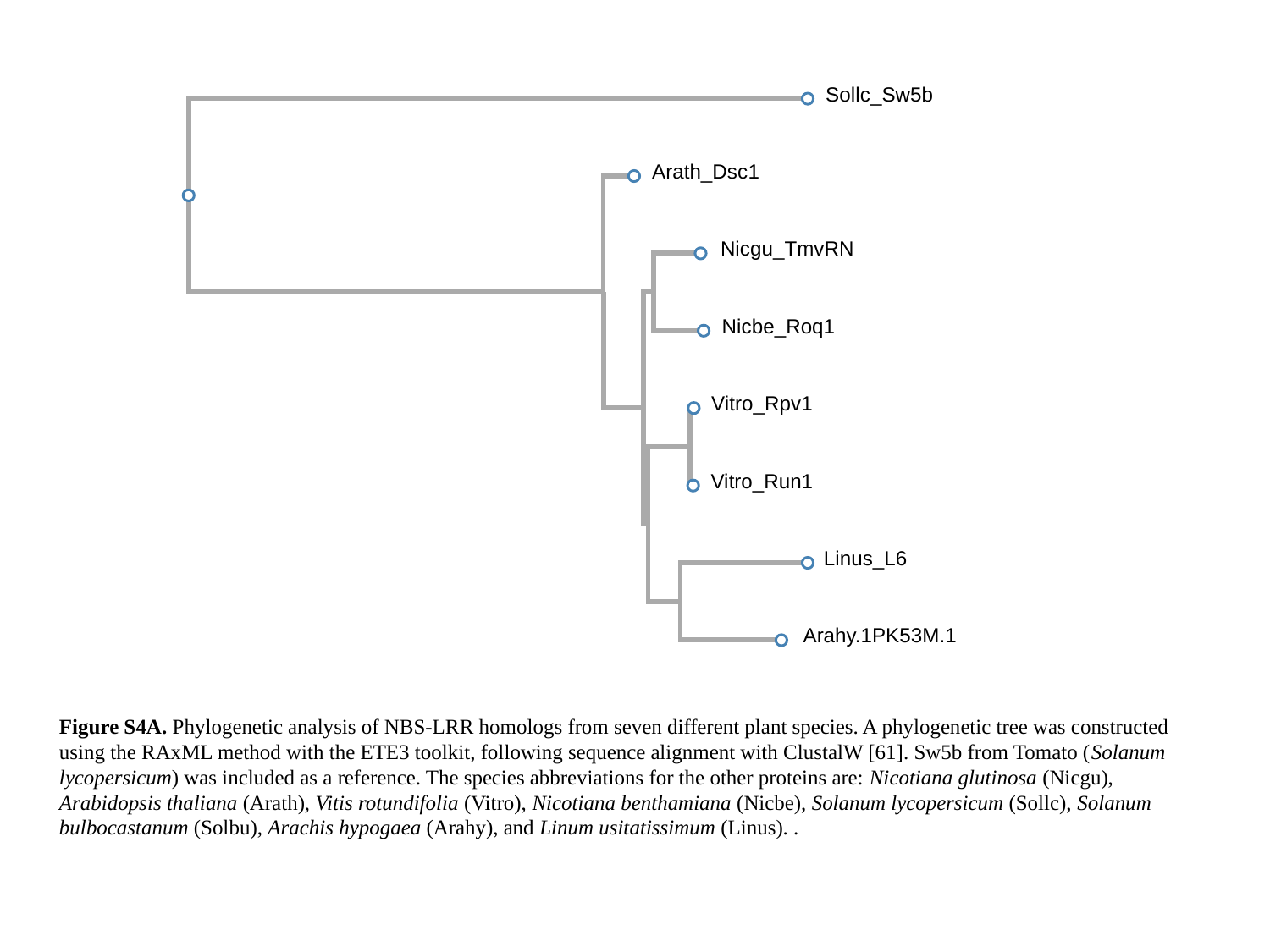

Sollc_Sw5b
Arath_Dsc1
Nicgu_TmvRN
Nicbe_Roq1
Vitro_Rpv1
Vitro_Run1
Linus_L6
Arahy.1PK53M.1
Figure S4A. Phylogenetic analysis of NBS-LRR homologs from seven different plant species. A phylogenetic tree was constructed using the RAxML method with the ETE3 toolkit, following sequence alignment with ClustalW [61]. Sw5b from Tomato (Solanum lycopersicum) was included as a reference. The species abbreviations for the other proteins are: Nicotiana glutinosa (Nicgu), Arabidopsis thaliana (Arath), Vitis rotundifolia (Vitro), Nicotiana benthamiana (Nicbe), Solanum lycopersicum (Sollc), Solanum bulbocastanum (Solbu), Arachis hypogaea (Arahy), and Linum usitatissimum (Linus). .

## Slide 6
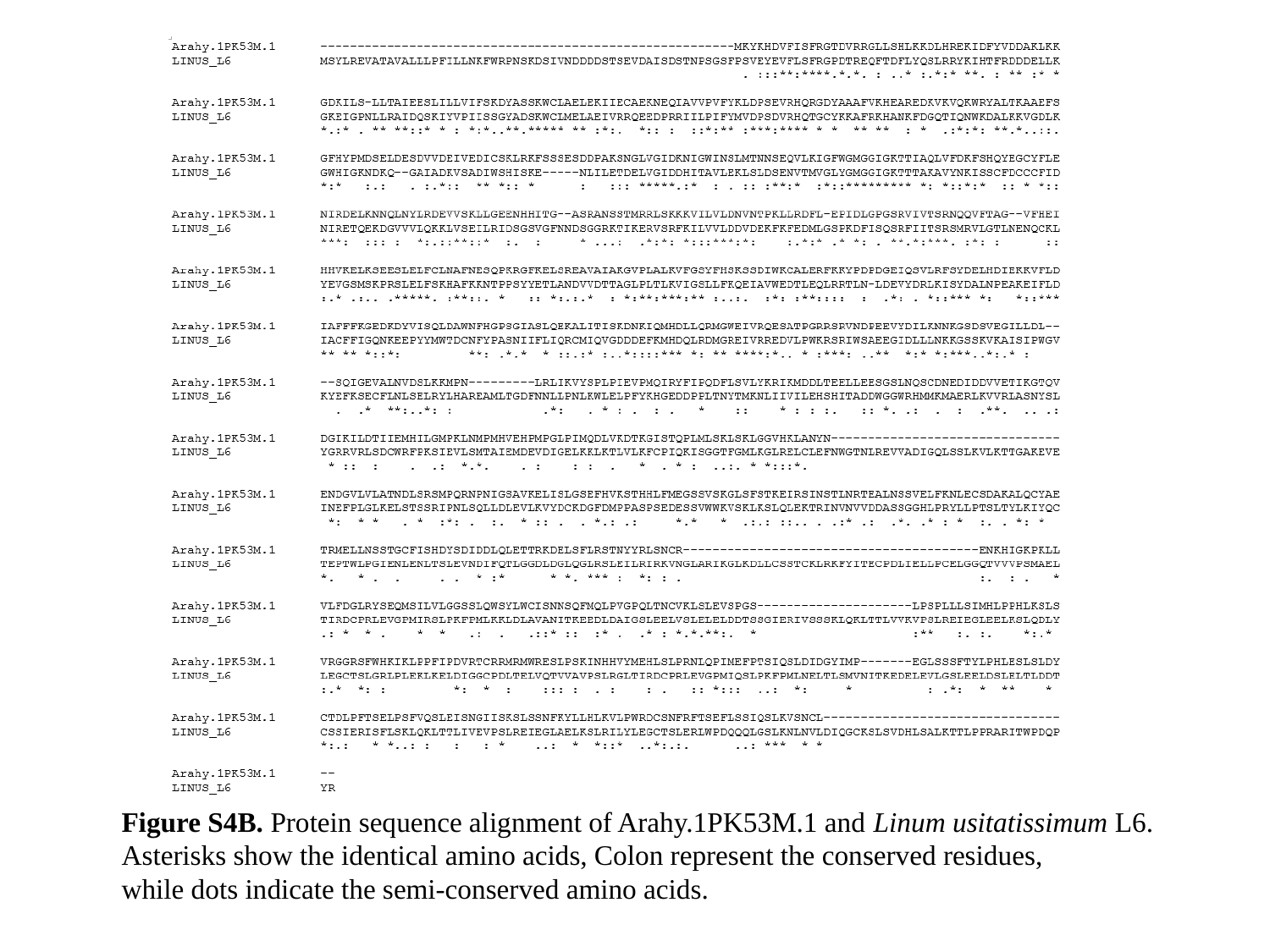

Figure S4B. Protein sequence alignment of Arahy.1PK53M.1 and Linum usitatissimum L6.
Asterisks show the identical amino acids, Colon represent the conserved residues,
while dots indicate the semi-conserved amino acids.

## Slide 7
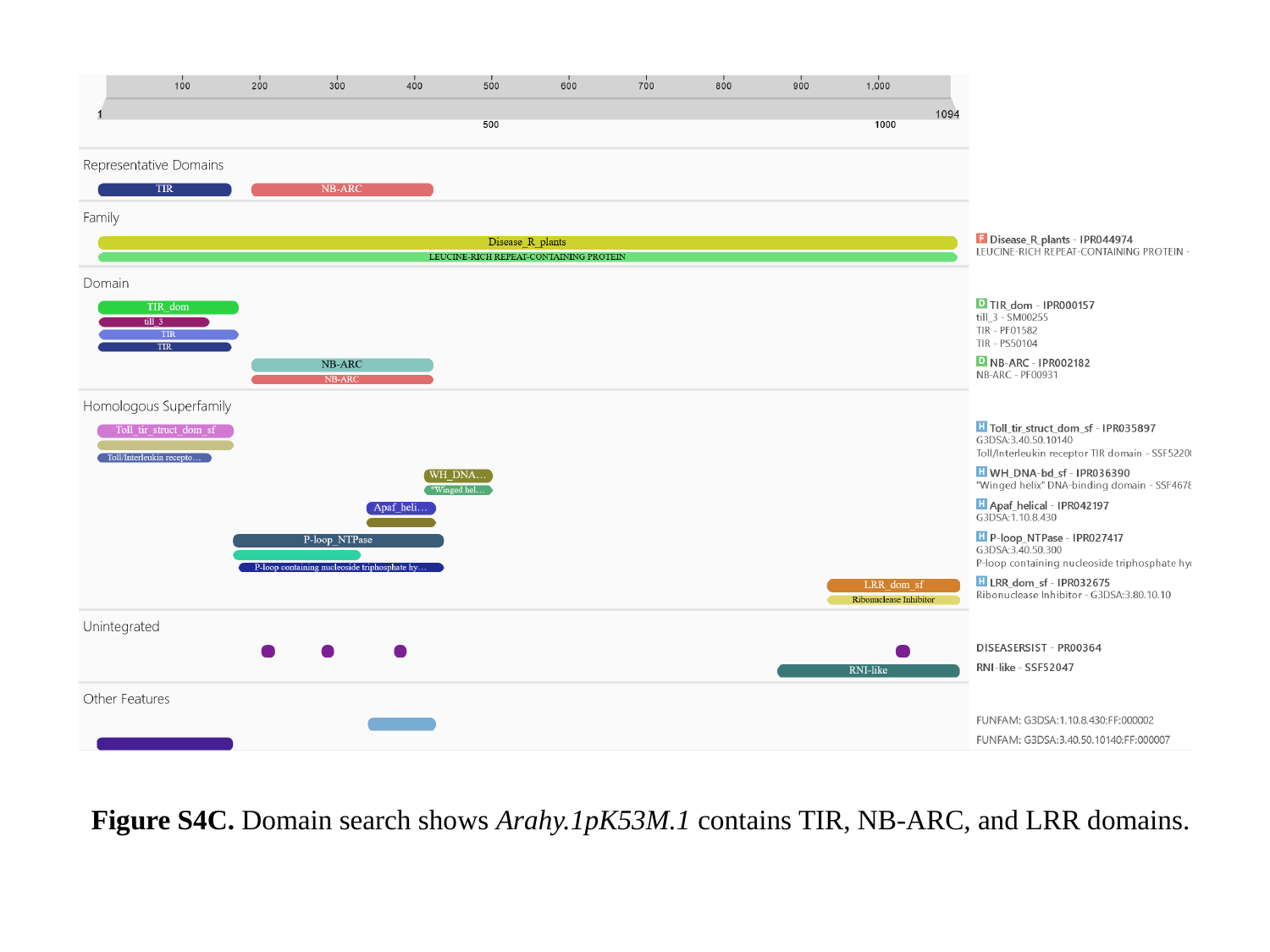

Figure S4C. Domain search shows Arahy.1pK53M.1 contains TIR, NB-ARC, and LRR domains.

## Slide 8
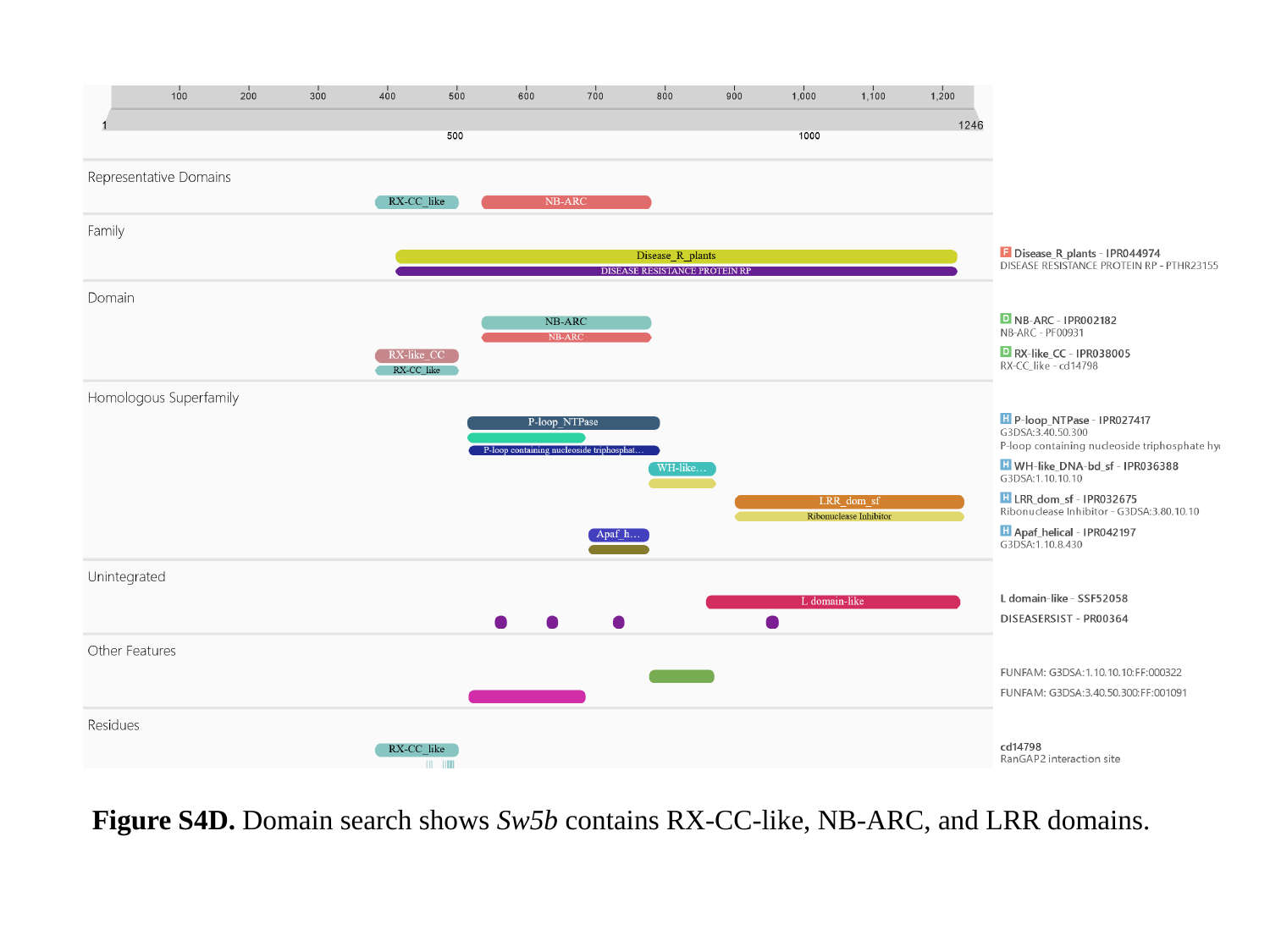

Figure S4D. Domain search shows Sw5b contains RX-CC-like, NB-ARC, and LRR domains.

## Slide 9
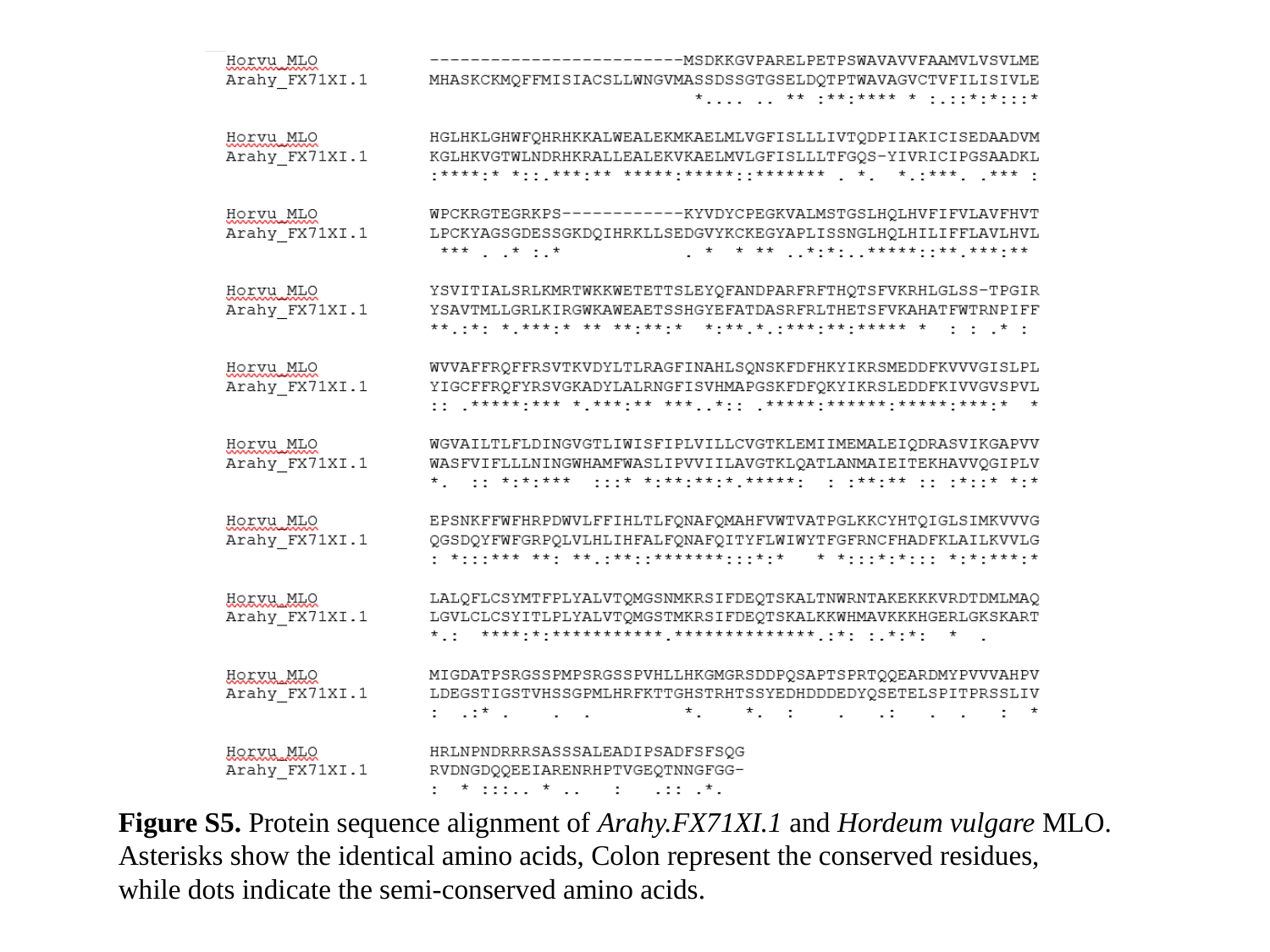

Figure S5. Protein sequence alignment of Arahy.FX71XI.1 and Hordeum vulgare MLO.
Asterisks show the identical amino acids, Colon represent the conserved residues,
while dots indicate the semi-conserved amino acids.
