# Supplementary material for: High-resolution genetic and physical mapping reveals a peanut spotted wilt disease resistance locus, PSWDR-1, to Tomato spotted wilt virus (TSWV), within a recombination cold-spot on chromosome A01
Source: BMC Genomics. 2025 Mar 6;26:224. doi: 10.1186/s12864-025-11366-7 (PMC11887336; doi:10.1186/s12864-025-11366-7)
Supplement: Supplementary file 2 — Supplementary Material 2. [file 12864_2025_11366_MOESM2_ESM.docx]

**Table S1** Summary of genetic linkage map construction using 143 RILs, Version I SNP Array, and SSR markers.

| **Linkage group** | **Distance (cM)** | **Number of loci** | **Map density (cM/loci)** |
| --- | --- | --- | --- |
| **A sub-genome linkage groups** | | | |
| A01 | 61.903 | 31 | 1.99 |
| A02 | 196.975 | 56 | 3.52 |
| A03 | 178.282 | 59 | 3.02 |
| A04 | 114.991 | 61 | 1.89 |
| A05 | 160.642 | 64 | 2.51 |
| A06 | 394.86 | 121 | 3.26 |
| A07 | 156.459 | 50 | 3.13 |
| A08 | 221.381 | 30 | 7.38 |
| A09 | 167.389 | 59 | 2.84 |
| A10 | 61.215 | 17 | 3.60 |
| **B sub-genome linkage groups** | | | |
| B01 | 149.488 | 57 | 2.62 |
| B02 | 170.391 | 45 | 3.79 |
| B03 | 192.634 | 48 | 4.01 |
| B04 | 80.532 | 53 | 1.52 |
| B05 | 263.953 | 46 | 5.74 |
| B06 | 150.652 | 47 | 3.21 |
| B07 | 346.329 | 73 | 4.74 |
| B08 | 203.396 | 53 | 3.84 |
| B09 | 107.604 | 32 | 3.36 |
| B10 | 1.873 | 10 | 0.19 |
| Total/mean | 3380.95 | 1012 | 3.34 |

**Table S2.** Identification of QTLs for resistance to Tomato spotted wilt virus (TSWV) using version 1 peanut SNP Array and SSR markers.

| **QTL** | **LG** | **Year** | **Flanking marker** | **Genetic interval** | **Length**  **(cM)** | **ADD** | **LOD** | **PVE (%)** |
| --- | --- | --- | --- | --- | --- | --- | --- | --- |
| *qTSWV1* | A01 | 2013 | *A01_9,459,521-* *A01_9,154,456* | 27.248- 30.86 | 3.612 | 0.7708 | 16.7242 | 41.4258 |
|  |  | 2011 |  |  |  | 0.3403 | 4.5458 | 14.0926 |
| *qTSWV2* | A01 | 2010 | *A01_9974460- SSRp23* | 42.417-46.812 | 4.395 | 0.3995 | 4.5214 | 15.1923 |

LOD: the logarithm of odds score, PVE: the phenotypic variance explained by individual QTL, ADD: the additive effect value
